# The influence of right ventricular stimulation on acute response to cardiac resynchronisation therapy

**DOI:** 10.1007/s12471-015-0770-x

**Published:** 2015-12-09

**Authors:** L. Wu, G.J. de Roest, M.L. Hendriks, A.C. van Rossum, C.C. de Cock, C.P. Allaart

**Affiliations:** Department of Cardiology, VU University Medical Center, Amsterdam, and Institute for Cardiovascular Research, Amsterdam, The Netherlands

**Keywords:** Right ventricular stimulation, Biventricular stimulation, Haemodynamic, Right ventricular function, Cardiac magnetic resonance imaging

## Abstract

**Background:**

The contribution of right ventricular (RV) stimulation to cardiac resynchronisation therapy (CRT) remains controversial. RV stimulation might be associated with adverse haemodynamic effects, dependent on intrinsic right bundle branch conduction, presence of scar, RV function and other factors which may partly explain non-response to CRT. This study investigates to what degree RV stimulation modulates response to biventricular (BiV) stimulation in CRT candidates and which baseline factors, assessed by cardiac magnetic resonance imaging, determine this modulation.

**Methods and results:**

Forty-one patients (24 (59 %) males, 67 ± 10 years, QRS 153 ± 22 ms, 21 (51 %) ischaemic cardiomyopathy, left ventricular (LV) ejection fraction 25 ± 7 %), who successfully underwent temporary stimulation with pacing leads in the RV apex (RV_apex_) and left ventricular posterolateral (PL) wall were included. Stroke work, assessed by a conductance catheter, was used to assess acute haemodynamic response during baseline conditions and RV_apex_, PL (LV) and PL+RV_apex_ (BiV) stimulation.

Compared with baseline, stroke work improved similarly during LV and BiV stimulation (∆+ 51 ± 42 % and ∆+ 48 ± 47 %, both *p* < 0.001), but individual response showed substantial differences between LV and BiV stimulation. Multivariate analysis revealed that RV ejection fraction (β = 1.01, *p* = 0.02) was an independent predictor for stroke work response during LV stimulation, but not for BiV stimulation. Other parameters, including atrioventricular delay and scar presence and localisation, did not predict stroke work response in CRT.

**Conclusion:**

The haemodynamic effect of addition of RV_apex_ stimulation to LV stimulation differs widely among patients receiving CRT. Poor RV function is associated with poor response to LV but not BiV stimulation.

**Electronic supplementary material:**

The online version of this article (doi:10.1007/s12471-015-0770-x) contains supplementary material, which is available to authorized users.

## Introduction

Cardiac resynchronisation therapy (CRT) is an established therapy for drug-refractory symptomatic heart failure patients with reduced left ventricular ejection fraction (LVEF) and left bundle branch block (LBBB) [1]. Benefit from CRT depends on patient-specific factors such as electrical and mechanical dyssynchrony, scar tissue and viability [2–4]. Moreover, left ventricular (LV) lead position significantly influences CRT response [4]. The effect of LV-only stimulation has been studied intensively, whereas the influence of right ventricular (RV) stimulation in CRT has received less attention and remains controversial [5]. In general, CRT with and without RV stimulation (biventricular (BiV) and LV stimulation, respectively) show similar effects in acute haemodynamic studies [6] and also in studies using clinical and echocardiographic parameters during long-term follow-up [5]. In individual patients, however, both improvement and deterioration of pump function have been reported when RV stimulation was added to LV stimulation [6, 7]. Mechanisms underlying this phenomenon are still poorly understood. Hypotheses include a beneficial effect of intrinsic right branch bundle (RBB) conduction in combination with LV stimulation as opposed to BiV stimulation [8, 9] and effects of the location of scar [2, 10]. In addition, modelling studies suggest a significant role for the right ventricle [11].

The aim of the present study is to investigate to what degree RV stimulation modulates response to BiV stimulation and which factors determine this modulation. Acute haemodynamic response was assessed during RV, LV and BiV stimulation, using pressure-volume loops (PV loops) and related to baseline variables including PQ-time, volumes assessed by cardiovascular magnetic resonance (CMR) imaging, ejection fractions and scar.

## Methods

### Study population

Patients were selected from the Temporary Biventricular Stimulation database, which evaluated acute haemodynamic response using PV loops in patients with mild to advanced heart failure (New York Heart Association Class II–III), severely depressed LVEF (≤ 35 %), sinus rhythm, and at least 3 months of stable optimally tolerated medical therapy [12]. Since QRS width was not an inclusion criterion, the database contains patients with narrow QRS, and right and left bundle branch block. For the present study, patients were selected when both PV loops and CMR scan were suitable for analysis, and the electrocardiogram showed LBBB according to the AHA/ACCF/HRS criteria [13]. When criteria developed by Strauss et al. were satisfied, LBBB was designated as ‘stringent’, otherwise as ‘lenient’ [14]. All patients provided written informed consent before the study procedures.

### Cardiac magnetic resonance imaging and analysis

CMR studies were performed on a 1.5-Tesla whole body scanner (Magnetom Sonata, Siemens, Erlangen, Germany), using six-channel phased-array body coils, as described earlier [12]. Steady-state free precession cines were acquired in a single breath-hold during mild expiration for 8–10 s (slice thickness 5 mm, slice gap 5 mm, temporal resolution < 50 ms, repetition time 3.2 ms, echo time 1.54 ms, flip angle 60° and a typical image resolution of 1.3 × 1.6 mm. The number of phases within the cardiac cycle was set at 20).

CMR images were analysed offline, using MASS analysis software (Medis, Leiden, the Netherlands). Endocardial borders were drawn semi-automatically in a short-axis stack to compute LV volumes, LVEF, RV volumes and RV ejection fraction (RVEF). Furthermore, the quantity of myocardial scar tissue was analysed using the Full-Width-at-Half-Maximum technique and presented as a percentage of the LV mass derived from the cine images [15]. The 17-segment American Heart Association model was used for segmental scar tissue analysis.

### Temporary stimulation procedure

In a separate procedure prior to CRT implantation, invasive haemodynamic measurements were obtained at baseline and during temporary stimulation. Temporary stimulation was accomplished by placing temporary pacing leads in the RV apex (RV_apex_), a posterolateral (PL) vein at the mid-ventricular level and in a subgroup of patients RV septum (RV_septal_), as previously described [12]. Stimulation was performed with an atrioventricular delay of 100 ms, and an interventricular interval of 0 ms. The following stimulation configurations were used: RV_apex_, PL (LV) and PL+RV_apex_ (BiV) stimulation. In a subgroup of patients, also RV_septal_ and PL+RV_septal_ (BiV_sept_) stimulation was performed.

Haemodynamic parameters were obtained by conductance catheter (CD Leycom, Leiden, the Netherlands) measurements enabling PV loop reconstruction, as described previously (Fig. 1, [12, 16]). Baseline measurements were acquired prior to and after every pace configuration. Each measurement consisted of 40–60 representative heartbeats, these were averaged and used for offline analysis. The effect of each stimulation configuration was calculated as the relative change compared with the mean of the adjacent baselines. LV stroke work was used to assess acute haemodynamic outcome. LVdP/dt_max_ was used as a second outcome measure. Results are shown in the Appendix.

**Figure:**
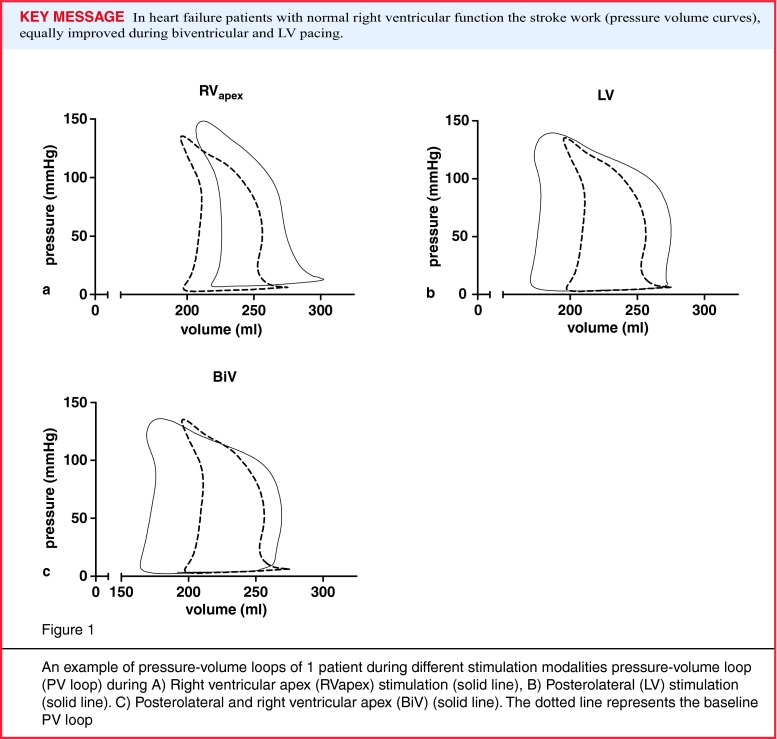

### Statistical methods

Statistical analysis was performed with IBM SPSS Statistics for Windows, Version 20.0. Continuous variables are expressed as mean ± standard deviation. All variables were tested for normal distribution using the Kolmogorov-Smirnov test, the Shapiro-Wilk test was used for the subgroups. Invasive haemodynamic variables were compared using repeated ANOVA or the Wilcoxon ranked test, both with Bonferroni correction, for normal and non-normal distribution respectively. Univariate regression analyses were used to determine the relationship between age, gender, clinical status, cardiac function and change in LV stroke work. Variables with *p* < 0.05 were entered into the multivariable regression analysis using backward elimination to identify independent predictors for change in LV stroke work. Pearson correlation was calculated between baseline PQ-time and haemodynamic response during stimulation. The independent non-parametric test was used to study the difference in the presence of scar tissue in the four LV walls between patients who respond better to LV stimulation and those who respond better to BiV stimulation. A *p* < 0.05 was considered significant.

## Results

Forty-one patients were selected from the original database (*n* = 88). Baseline characteristics are given in Table 1. In a subgroup of 21 patients RV_septal_ stimulation was performed. Data using LVdP/dt_max_ as an outcome measure are presented in the Appendix.

**Table 1:** Baseline characteristics

Baseline characteristics	*n* = 41
Age (years)	67 ± 10
Male	24 (59 %)
QRS (ms)	153 ± 22
Stringent LBBB (n)	28
Lenient LBBB (n)	13
Ischaemic, n	21 (51 %)
NYHA class, n	
I/II/III/I	0/8/33/0
NT-pro BNP (ng/L)^a^	1509 ± 1318
RVEDV (ml)	153 ± 53
RVESV (ml)	90 ± 48
RVEF (%)	43 ± 16
TAPSE (mm)	18 ± 7
LVEDV (ml)	277 ± 90
LVESV (ml)	211 ± 81
LVEF (%)	25 ± 8
LVSW (L·mmHg)	5.5 ± 2.5
LVdP/dt_max_ (mmHg/s)	868 ± 182
Scar (%)^b^	8.9 ± 11.5

Stringent left bundle branch block (*LBBB*) according to Strauss criteria (see Methods), lenient LBBB according to AHA/ACCF/HRS criteria (see Methods), *NYHA class* New York Heart Association functional class, *NT-pro-BNP* N-terminal prohormone of brain natriuretic peptide, *RVEDV* right ventricular end-diastolic volume, *RVESV* right ventricular end-systolic volume, *RVEF* right ventricular ejection fraction, *TAPSE* tricuspid annular plane systolic excursion, *LVEDV* left ventricular end-diastolic volume, *LVESV* left ventricular end-systolic volume, *LVEF* left ventricular ejection fraction, *LVSW* left ventricular stroke work, *LVdP/dt*_*max*_ left ventricular dP/dt_max_.

^a^3 missing.

^b^15 missing.

### Haemodynamic measurements

**Fig. 2:**
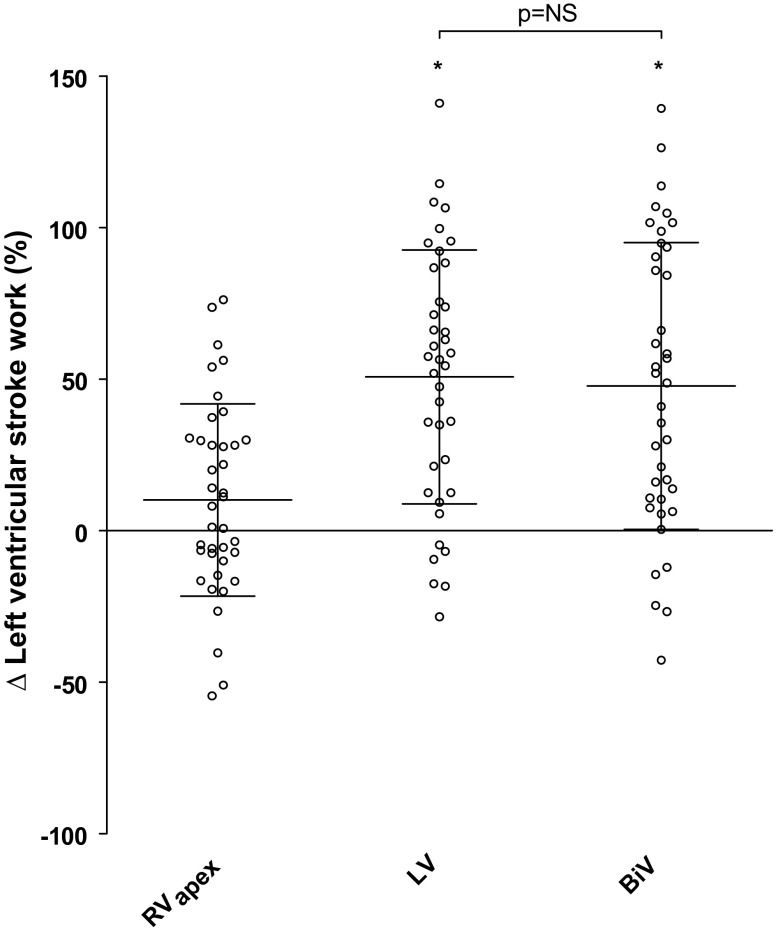
Acute effect of different stimulation modalities on left ventricular stroke work compared with baseline. The stimulation modalities (right ventricular apex (*RV*_*apex*_), posterolateral (*LV*), posterolateral and right ventricular apex stimulation (BiV)) versus left ventricular stroke work response (% change compared with baseline). * p < 0.001, compared with baseline

Acute haemodynamic responses of RV_apex_, LV and BiV stimulation (∆+ 10 ± 32 %, ∆+ 51 ± 42 % and ∆+ 48 ± 47 %, respectively) are presented in Fig. 2. Compared with baseline, both LV and BiV stimulation were associated with a significant increase in LV stroke work (both *p* < 0.001), while RV_apex_ pacing was not. However, no significant difference in LV stroke work increase was observed between LV and BiV stimulation (*p* = 1.00).

In the RV_septal_ subgroup, RV_septal_ stimulation did not result in a significant increase in LV stroke work (∆+ 3 ± 19, *p* = 1.00) while BiV_sept_ stimulation was associated with a significant rise in LV stroke work compared with baseline (∆+ 32 ± 39 %, *p* < 0.01). No differences in LV stroke work were found between stimulation at RV_apex_ and RV_septal_ (∆+ 9 ± 34 % vs ∆+ 3 ± 19 %, *p* = 1.00, respectively), LV and BiV_sept_ (∆+ 41 ± 43 % vs ∆+ 32 ± 39 %, *p* = 0.06, respectively) and BiV and BiV_sept_ (∆+ 42 ± 52 % vs ∆+ 32 ± 39 %, *p* = 1.00, respectively).

### The effect of right ventricular stimulation on CRT

**Fig. 3:**
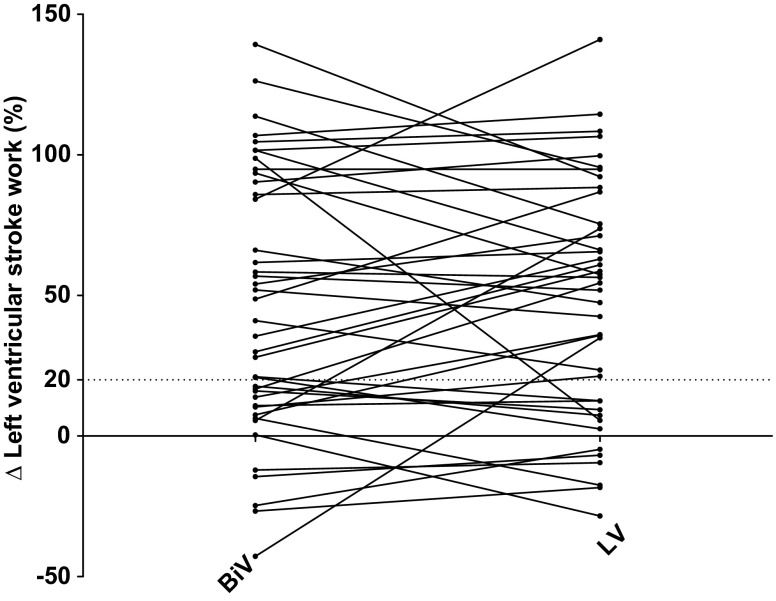
Individual left ventricular stroke work effect of switching off RV stimulation.

There was a substantial variation in individual LV stroke work response during BiV and LV stimulation, as shown in Fig. 3. Compared with BiV stimulation, LV stroke work improved during LV stimulation in 18 patients, whereas it decreased in 15 patients. No difference (< 10 % variation) in LV stroke work response between BiV stimulation and LV stimulation was found in only 8 patients.

**Fig. 4:**
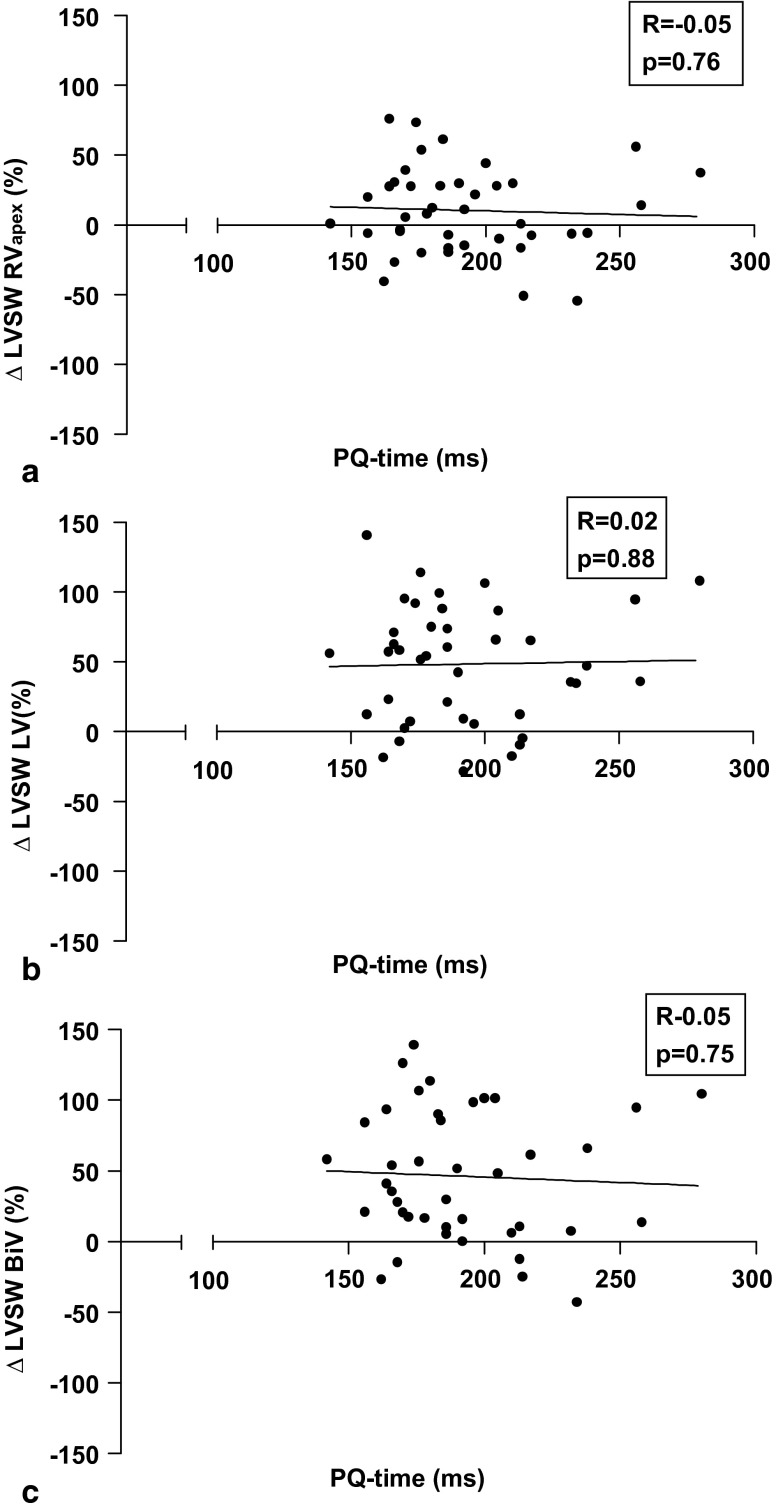
Correlation between baseline PQ-time and left ventricular stroke work response. **a** Right ventricular apex (*RV*_*apex*_) stimulation, **b** Posterolateral (*LV*) stimulation and **c** Posterolateral and right ventricular apex (*BiV*) stimulation. No significant relations were found

To evaluate the effect of intrinsic RBB conduction in CRT, PQ-time were related to LV stroke work response during various stimulation modalities (Fig. 4). No significant effects of PQ-time on LV stroke work response were found. To further evaluate determinants of response to RV_apex_, LV and BiV stimulation, regression analysis was performed using clinical, haemodynamic and CMR parameters as presented in Table 2. Multivariate regression analysis showed that LV stroke work response both during RV_apex_ stimulation and during LV stimulation were significantly correlated with baseline RVEF. LV stroke work response during BiV stimulation, however, was correlated to baseline LVEF and not to RVEF. Further analysis showed that there is no baseline parameter that correlates with difference in LV stroke work during BiV and LV stimulation.

**Table 2:** Univariate and multivariate analysis of baseline parameters as predictor for left ventricular stroke work response during RVapex stimulation, LV, BiV stimulation and the difference between BiV and LV stimulation

	∆LVSW RV_apex_ Univariate		∆LVSW RV_apex_ Multivariate		∆LVSW LV Univariate		∆LVSW LV Multivariate		∆LVSW BiV Univariate		∆LVSW BiV Multivariate		∆LVSW BiV—LV Univariate	
	β	p	β	p	β	p	β	p	β	p	β	p	β	P
Ischemic (n/y)	7.48	0.45			− 13.53	0.31			5.61	0.71			15.80	0.07
Stringent LBBB (n/y)	10.44	0.34			21.51	0.13			10.39	0.51			− 5.72	0.55
PQ-time (ms)	− 0.05	0.76			0.03	0.88			− 0.08	0.75			− 0.12	0.43
Scar (%)	− 0.27	0.68			− 1.23	0.27			− 0.97	0.34			0.26	0.66
Septal scar (n/y)	− 3.67	0.78			− 11.49	0.29			− 2.54	0.89			− 11.49	0.29
PL scar (n/y)	− 0.93	0.93			8.87	0.54			− 2.27	0.89			− 8.64	0.37
RVEDV (ml)	− 0.09	0.34			− 0.28	0.02			− 0.24	0.09			0.07	0.44
RVESV (ml)	− 0.19	0.07			− 0.40	< 0.01			− 0.39	0.01			0.05	0.60
RVEF (%)	0.64	0.04	0.68	0.02	1.03	0.01	1.01	0.02	1.23	< 0.01	0.61	0.25	0.05	0.86
LVEDV (ml)	− 0.01	0.86			− 0.16	0.04			− 0.15	0.07			0.02	0.62
LVESV (ml)	− 0.04	0.53			− 0.39	0.02			− 0.20	0.08			0.02	0.76
LVEF (%)	1.14	0.06	0.38	0.63	1.88	0.03	0.89	0.44	2.76	< 0.01	2.76	< 0.01	0.58	0.26
LVSW (L·mmHg)	− 1.08	0.60			− 2.66	0.34			− 2.36	0.45			− 1.03	0.59
LVdP/dt_max_ (mmHg/s)	0.02	0.52			0.03	0.35			0.05	0.22			< 0.01	0.88

Left bundle branch block (*LBBB*), stringent LBBB according to Strauss criteria (see Methods), *PL* posterolateral, *RVEDV* right ventricular end-diastolic volume, *RVESV* right ventricular end-systolic volume, *RVEF* right ventricular ejection fraction, *LVEDV* left ventricular end-diastolic volume, *LVESV* left ventricular end-systolic volume, *LVEF* left ventricular ejection fraction, *LVSW* left ventricular stroke work, *LVdP/dt*_*max*_ left ventricular dP/dt_max_, *RV*_*apex*_, right ventricular apex, *LV* left ventricular, *BiV* biventricular.

Model multivariate analysis ∆LVSW RV_apex_: RVEF and LVEF.

Model multivariate analysis ∆LVSW LV: RVEF and LVEF.

Model multivariate analysis ∆LVSW BiV: RVEF and LVEF.

### The effect of scar location on CRT

Scar analysis was performed in 26 patients, but scar tissue was present in only 14 patients. Segmental scar analysis was performed in these 14 patients, in total 238 segments. Patients with scar tissue were divided into patients who positively responded to LV stimulation (*n* = 10) and those who positively responded to BiV stimulation (*n* = 4). Table 3 shows the amount of scar tissue in the 4 LV walls. No significant differences in the amount of scar tissue were found between patients who responded better to LV stimulation and those who responded better to BiV stimulation.

**Table 3:** The relation between scar percentage and left ventricular stroke work change during LV and BiV stimulation

Scar (%)	Anterior wall	Septal wall	Inferior wall	Lateral wall
**LV better**	19.9 ± 22.3 %	8.9 ± 7.8 %	6.7 ± 5.8 %	9.8 ± 8.7 %
**BiV better**	39.9 ± 29.7 %	26.0 ± 18.6 %	12.1 ± 13.0 %	8.4 ± 6.4 %
*p* **-value**	0.20	0.16	0.48	0.72

*LV* left ventricular, *BiV* biventricular.

## Discussion

This study shows that BiV and LV stimulation elicit comparable acute haemodynamic benefit, supporting the results of earlier acute haemodynamic and long-term follow-up studies [6, 7, 17, 18]. In the individual patient, however, haemodynamic response to BiV and LV stimulation might differ substantially. Several patients were found to change from non-responder to responder by addition of RV_apex_ stimulation to LV stimulation, but the reverse response was also observed. LV stroke work response to LV stimulation was significantly correlated with baseline RVEF, whereas LV stroke work response to BiV stimulation was not. PQ-time did not significantly affect LV stroke work response to either LV or BiV stimulation. Analyses using LVdP/dt_max_ as the outcome measure will be discussed in the Appendix.

### Determinants of response to stimulation

Previous studies suggested that intrinsic RBB conduction combined with LV stimulation (fusion pacing) might be superior to BiV stimulation in patients with normal PQ-intervals [8, 9]. Extrapolating this hypothesis to this study, one would expect addition of RV_apex_ stimulation to LV stimulation to be beneficial, especially in patients with long PQ-intervals, whereas in patients with normal PQ-intervals LV stimulation (with intrinsic RBB conduction) is expected to be superior. However, we found no relation between LV stimulation and the PQ-interval. Thus, intrinsic RBB conduction does not seem to play a significant role in the observed differences between LV and BiV stimulation. However, these results should be interpreted with caution, since atrioventricular delay was set at 100 ms, which will cause full capture of the LV using both LV and BiV stimulation in the majority of cases.

Mechanical properties were also evaluated and related to the effect of RV stimulation in CRT. RV stimulation per se is known to exert detrimental effects on pump function, especially in patients with heart failure [19, 20]. In the present study, a significant positive correlation between baseline RVEF and LV stroke work response during LV stimulation was found, whereas no relation was found between baseline RVEF and BiV stimulation. In an elegant modelling study, Lumens et al. showed that myofibre work distribution over the LV and RV myocardium is different during LV and BiV stimulation [11]. Their model suggested that LV stimulation is less effective than BiV stimulation in patients with a decreased RV contractile function, since myocardial work in this stimulation configuration is mainly generated in the interventricular septum and the RV free wall. This is confirmed in our study, we found a significant correlation between LV stroke work response and RVEF in LV but not in BiV stimulation. However, neither RVEF and LVEF were correlated with the difference in LV stroke work during BiV and LV stimulation, suggesting that these parameters should be used carefully to guide stimulation modality.

In addition, Lumens et al. suggested that patients with septal scar would respond better to BiV stimulation, since in their model, most myocardial work is generated in the anterior and inferior wall during BiV stimulation and pump function is thus relatively independent of the septal contribution [11]. This study could not confirm this notion in a relatively small patient group, although patients who respond better to BiV stimulation seem to have more septal scar tissue than those who respond better to LV stimulation (26.0 ± 18.6 % vs 8.9 ± 7.8 %, respectively, *p* = 0.16).

### Clinical consequences

Evaluation of haemodynamic consequences of the addition of RV to LV stimulation can be considered as a crude method of interventricular optimisation. Previous studies showed significant individual effects of interventricular optimisation in a substantial subset of patients, assessed by both invasive and noninvasive measures [21–23]. The results of the present study corroborate these findings. In a previous study we found a LV stroke work increase of > 20 % is a strong predictor for long-term response to CRT [24]. Applying that cut-off, 3 of 27 responders (10 %) during BiV stimulation became non-responders by switching to LV stimulation. Conversely, 6 of 15 non-responders (40 %) changed to LV stroke work responders by the same manoeuvre. No baseline parameter was correlated with the individual response to CRT, emphasising the need of an individualised approach to optimisation.

### Study limitations

Several limitations need to be addressed. First, this study contained a study population of only 41 patients. Since CRT response is multifactorially determined, this limited number of patients may result in factors exerting small effects remaining unrecognised. The scar analysis is performed on even smaller numbers and should be interpreted with caution. Secondly, PV loop assessment requires simultaneous acquisition of a pressure and a volume signal. Pressure signals are obtained from a solid state pressure sensor and are therefore reliable signals. Obtaining volume signals, however, requires careful catheter placement which can be very arduous, particularly in dilated ventricles. Repeated baseline measurements and elaborate data optimisation were used to ensure stable catheter position and data validity. Moreover, the outcome measure of this study (∆ LV stroke work) is a relative measure, decreasing the effect of systematic errors. Thirdly, the study protocol did not include atrioventricular optimisation. Previous studies have shown that atrioventricular and interventricular optimisation are interrelated, but the size of this interrelation is generally assumed to be small and contradictory results have been reported [21, 25]. On the other hand, we observed substantial effects of RV_apex_ stimulation on LV stroke work response. These effects showed no relation to baseline PQ interval, which renders major effects on the outcome of the present study less likely. Finally, this study focuses on acute response to CRT, without long-term follow-up with respect to clinical parameters. However, we recently demonstrated that LV stroke work response predicted long-term reverse remodelling with high accuracy [24].

#### Funding

None.

## Electronic supplementary material





## References

[1] Cleland JG, Daubert JC, Erdmann E (2005). The effect of cardiac resynchronization on morbidity and mortality in heart failure. N Engl J Med.

[2] Marsan NA, Westenberg JJ, Ypenburg C (2009). Magnetic resonance imaging and response to cardiac resynchronization therapy: relative merits of left ventricular dyssynchrony and scar tissue. Eur Heart J.

[3] Hawkins NM, Petrie MC, MacDonald MR (2006). Selecting patients for cardiac resynchronization therapy: electrical or mechanical dyssynchrony?. Eur Heart J.

[4] Saba S, Marek J, Schwartzman D (2013). Echocardiography-guided left ventricular lead placement for cardiac resynchronization therapy: results of the Speckle Tracking Assisted Resynchronization Therapy for Electrode Region (STARTER) Trial. Circ Heart Fail.

[5] Thibault B, Ducharme A, Harel F (2011). Left ventricular versus simultaneous biventricular pacing in patients with heart failure and a QRS complex ≥ 120 milliseconds. Circulation.

[6] Kass DA, Chen CH, Curry C (1999). Improved left ventricular mechanics from acute VDD pacing in patients with dilated cardiomyopathy and ventricular conduction delay. Circulation.

[7] Touiza A, Etienne Y, Gilard M (2001). Long-term left ventricular pacing: assessment and comparison with biventricular pacing in patients with severe congestive heart failure. J Am Coll Cardiol.

[8] van Gelder BM, Bracke FA, Meijer A (2005). The hemodynamic effect of intrinsic conduction during left ventricular pacing as compared with biventricular pacing. J Am Coll Cardiol.

[9] Vatasescu R, Berruezo A, Mont L (2009). Midterm ‘super-response’ to cardiac resynchronization therapy by biventricular pacing with fusion: insights from electro-anatomical mapping. Europace.

[10] Bleeker GB, Kaandorp TA, Lamb HJ (2006). Effect of posterolateral scar tissue on clinical and echocardiographic improvement after cardiac resynchronization therapy. Circulation.

[11] Lumens J, Ploux S, Strik M (2013). Comparative electromechanical and hemodynamic effects of left ventricular and biventricular pacing in dyssynchronous heart failure: electrical resynchronization versus left-right ventricular interaction. J Am Coll Cardiol.

[12] de Roest GJ, Allaart CP, de Haan S (2011). Effects of QRS duration and pacing location on pressure-volume loop evaluation of cardiac resynchronization therapy in end-stage heart failure. Am J Cardiol.

[13] Surawicz B, Childers R, Deal BJ (2009). AHA/ACCF/HRS recommendations for the standardization and interpretation of the electrocardiogram: part III: intraventricular conduction disturbances: a scientific statement from the American Heart Association Electrocardiography and Arrhythmias Committee, Council on Clinical Cardiology; the American College of Cardiology Foundation; and the Heart Rhythm Society. Endorsed by the International Society for Computerized Electrocardiology. J Am Coll Cardiol.

[14] Strauss DG, Selvester RH (2009). The QRS complex–a biomarker that ‘images’ the heart: QRS scores to quantify myocardial scar in the presence of normal and abnormal ventricular conduction. J Electrocardiol.

[15] Flett AS, Hasleton J, Cook C (2011). Evaluation of techniques for the quantification of myocardial scar of differing etiology using cardiac magnetic resonance. JACC Cardiovasc Imaging.

[16] Kass DA (1992). Clinical evaluation of left heart function by conductance catheter technique. Eur Heart J.

[17] Auricchio A, Stellbrink C, Block M (1999). Effect of pacing chamber and atrioventricular delay on acute systolic function of paced patients with congestive heart failure. The Pacing Therapies for Congestive Heart Failure Study Group. The Guidant Congestive Heart Failure Research Group. Circulation.

[18] Damy T, Ghio S, Rigby AS (2013). Interplay between right ventricular function and cardiac resynchronisation therapy: an analysis of the CARE-HF trial. J Am Coll Cardiol.

[19] Lieberman R, Padeletti L, Schreuder J (2006). Ventricular pacing lead location alters systemic hemodynamics and left ventricular function in patients with and without reduced ejection fraction. J Am Coll Cardiol.

[20] Sweeney MO, Hellkamp AS (2006). Heart failure during cardiac pacing. Circulation.

[21] Abraham WT, Leon AR, St John Sutton MG (2012). Randomized controlled trial comparing simultaneous versus optimized sequential interventricular stimulation during cardiac resynchronization therapy. Am Heart J.

[22] Barold SS, Ilercil A, Herweg B (2008). Echocardiographic optimization of the atrioventricular and interventricular intervals during cardiac resynchronization. Europace.

[23] Leon AR, Abraham WT, Brozena S (2005). Cardiac resynchronization with sequential biventricular pacing for the treatment of moderate-to-severe heart failure. J Am Coll Cardiol.

[24] de Roest GJ, Allaart CP, Kleijn SA (2013). Prediction of long-term outcome of cardiac resynchronization therapy by acute pressure-volume loop measurements. Eur J Heart Fail.

[25] Porciani MC, Dondina C, Macioce R (2005). Echocardiographic examination of atrioventricular and interventricular delay optimization in cardiac resynchronization therapy. Am J Cardiol.

