# Supplementary material for: The influence of right ventricular stimulation on acute response to cardiac resynchronisation therapy
Source: Neth Heart J. 2015 Dec 9;24(1):66–72. doi: 10.1007/s12471-015-0770-x (PMC4692833; doi:10.1007/s12471-015-0770-x)
Supplement: Supplementary file 1 [file 12471_2015_770_MOESM1_ESM.docx]

**APPENDIX**

**The influence of right ventricular stimulation on acute response to cardiac resynchronisation therapy**

This appendix presents data on the effects of RV_apex_, LV and BiV stimulation, using LVdP/dt_max_ as the outcome measure instead of LV stroke work. Data were derived from exactly the same patient population and experiments. For further details see the Methods section of the main paper. In addition, LVdP/dt_max_ response is defined as 10% improvement compared with baseline [1].

**Hemodynamic measurements**

Acute haemodynamic responses of RV_apex_, LV and BiV stimulation are presented in Fig. 1. These results are similar to LV stroke work. However, no significant difference in LVdP/dt_max_ increase was observed between LV and BiV stimulation. There is a significant increase in LVdP/dt_max_ during BiV_sept_ compared with baseline (Δ+9±10, p=0.001). In contrast to LV stroke work, there is a significant difference between LV and Biv_sept_ in favour of LV stimulation (∆+13±10% vs ∆+9±10%, p=0.024, respectively).

**The effect of right ventricular stimulation on CRT**

There was a substantial variation in individual LVdP/dt_max_ response during BiV and LV stimulation, as shown in Fig. 2. Compared with BiV stimulation, in 28 patients LVdP/dt_max_ improved during LV stimulation, whereas LVdP/dt_max_ decreased in 12 patients. No difference (<10% variation) in LVdP/dt_max_ response between BiV stimulation and LV stimulation was found in only 1 patient.

LVdP/dt_max_ response is defined as 10% improvement compared with baseline [1]. Applying that cut-off, 1 of 22 responders (5%) during BiV stimulation became non-responders by switching to LV stimulation. Conversely, 5 of 19 non-responders (26%) changed to responders by the same manoeuvre.

To evaluate the effect of intrinsic RBB conduction in CRT, PQ-time was related to LVdP/dt_max_ response during various stimulation modalities (Fig. 3). No significant effects of PQ-time on LVdP/dt_max_ response were found. To further evaluate determinants of response to RV_apex_, LV and BiV stimulation, regression analysis was performed using clinical, haemodynamic and CMR parameters as presented in Table 1. Multivariate regression analysis showed that a stringent LBBB is significantly correlated with LVdP/dt_max_ response during RV_apex_ stimulation, this was not found during other stimulation modalities.

**Conclusion**

In summary, the numbers of patients improving by RV, LV and BiV stimulation are comparable, although the magnitude of the response (in percentages) is smaller. The same applies for changes when switching from BiV to LV stimulation. However, when comparing the effects of stimulation on changes in LVdP/dt_max_ and LV stroke work in the individual patient, no significant correlation was found. In a previous study comparing these two outcome measures, we showed that acute LV stroke work improvement is superior in predicting long-term outcome compared with LVdP/dt_max_ [2] explaining our present choice. Based on this we feel that the results using LVdP/dt_max_ as the outcome measure should be interpreted with extreme caution.

**Figure 1: Acute effect of different stimulation modalities on LVdP/dt_max_ compared with baseline.**

The stimulation modalities (right ventricular apex (RV_apex_), posterolateral (LV), posterolateral and right ventricular apex stimulation (BiV)) versus left ventricular stroke work response (% change compared with baseline).

* = p<0.001, compared with baseline

**Figure 2: Individual LVdP/dt_max_ effect of switching off RV stimulation.**

Dot-line plot showing left ventricular stroke work response during biventricular (BiV) stimulation and posterolateral (LV) stimulation for individual patients. The dotted line represents the cut-off for non-response, defined as a left ventricular dP/dt_max_  improvement of ≤ 10% [1].

**Figure 3: Correlation between baseline PQ-time and left ventricular LVdP/dt_max_.**

A) Right ventricular apex (RV_apex_) stimulation, B) Posterolateral (LV) stimulation and C) Posterolateral and right ventricular apex (BiV) stimulation. No significant relations were found.

|  | ***∆LVdP/dt_max_ RV_apex_***  ***Univariate***  ***β p*** | | ***∆LVdP/dt_max_ RV_apex_***  ***Multivariate***  ***β p*** | | ***∆LVdP/dt_max_ LV***  ***Univariate***  ***β p*** | | ***∆LVdP/dt_max_ LV***  ***Multivariate***  ***β p*** | | ***∆LVdP/dt_max_ BiV***  ***Univariate***  ***β p*** | | ***∆LVdP/dt_max_ BiV***  ***Multivariate***  ***β p*** | | ***∆LVdP/dt_max_ BiV - LV***  ***Univariate***  ***β p*** | |
| --- | --- | --- | --- | --- | --- | --- | --- | --- | --- | --- | --- | --- | --- | --- |
| Ischaemic (n/y) | -1.43 | 0.55 |  |  | -6.1 | 0.11 |  |  | -8.50 | 0.17 |  |  | -2.41 | 0.62 |
| Stringent LBBB (n/y) | 7.48 | <0.01 | 6.87 | <0.01 | 12.21 | <0.01 | 7.46 | 0.24 | 14.92 | 0.02 | 8.77 | 0.30 | 2.72 | 0.60 |
| PQ-time (ms) | <-0.01 | 0.95 |  |  | -0.02 | 0.71 |  |  | -0.10 | 0.36 |  |  | -0.07 | 0.37 |
| Scar (%) | -0.24 | 0.14 |  |  | -0.65 | 0.05 | -0.55 | 0.09 | -0.83 | 0.05 | -0.83 | 0.05 | -0.19 | 0.39 |
| Septal scar (n/y) | -2.81 | 0.34 |  |  | -5.81 | 0.33 |  |  | -9.21 | 0.21 |  |  | -3.40 | 0.36 |
| PL scar (n/y) | -1.18 | 0.65 |  |  | -2.18 | 0.60 |  |  | -8.28 | 0.22 |  |  | -6.11 | 0.23 |
| RVEDV (ml) | -0.02 | 0.53 |  |  | 0.03 | 0.37 |  |  | 0.04 | 0.50 |  |  | <0.01 | 0.88 |
| RVESV (ml) | -0.04 | 0.16 |  |  | 0.05 | 0.21 |  |  | 0.03 | 0.64 |  |  | -0.02 | 0.70 |
| RVEF (%) | 0.15 | 0.04 | 0.09 | 0.24 | -0.12 | 0.34 |  |  | <-0.01 | 0.99 |  |  | 0.12 | 0.45 |
| LVEDV (ml) | 0.01 | 0.44 |  |  | 0.06 | <0.01 |  |  | 0.07 | 0.07 |  |  | <0.01 | 0.77 |
| LVESV (ml) | <0.01 | 0.76 |  |  | 0.07 | <0.01 |  |  | 0.09 | 0.04 |  |  | 0.02 | 0.59 |
| LVEF (%) | 0.23 | 0.14 |  |  | -0.45 | 0.08 | <0.01 | 0.99 | -0.85 | 0.05 | -0.58 | 0.24 | -0.40 | 0.22 |
| LVSW (L·mmHg) | 1.00 | 0.04 | 0.80 | 0.08 | 0.61 | 0.45 |  |  | 0.48 | 0.72 |  |  | -0.13 | 0.90 |
| LVdP/dt_max_ (mmHg/s) | <-0.01 | 0.83 |  |  | -0.02 | 0.05 | -0.02 | 0.09 | <-0.01 | 0.59 |  |  | 0.01 | 0.39 |

**Table 1:** Univariate and multivariate analysis of baseline parameters as predictor for left ventricular dP/dt_max_ response during RV_apex_ stimulation, LV, Biv stimulation and the difference between Biv and LV stimulation.

Left bundle branch block (LBBB), stringent LBBB according to Strauss criteria (see Methods), *PL* posterolateral, *RVEDV* right ventricular end-diastolic volume, *RVESV* right ventricular end-systolic volume, *RVEF* right ventricular ejection fraction, *LVEDV* left ventricular end-diastolic volume, *LVESV* left ventricular end-systolic volume, *LVEF* left ventricular ejection fraction, *LVSW* left ventricular stroke work, *LVdP/dt_max_* left ventricular dP/dt_max_, *RV_apex_*, right ventricular apex, *BiV* biventricular, *LV* left ventricular only

Model multivariate analysis ∆ LVdP/dt_max_ RV_apex_: RVEF, LVSW and stringent LBBB.

Model multivariate analysis ∆ LVdP/dt_max_ LV: Scar, LVEF, LVdP/dt_max_ and stringent LBBB

Model multivariate analysis ∆ LVdP/dt_max_ Biv: Scar, LVEF and stringent LBBB.

Multivariate regression analysis shows that baseline LVSW is significantly correlated with LVdP/dt_max_ response during RV_apex_ stimulation, however, this result was not found during the other pacing modalities. Further, the amount of scar seem to be significantly correlated with LVdP/dt_max_ response to Biv stimulation. Since the presence of scar is very little, this result should be interpreted with caution.

Reference List

1 Ginks MR, Duckett SG, Kapetanakis S, et al. Multi-site left ventricular pacing as a potential treatment for patients with posterolateral scar: insights from cardiac magnetic resonance imaging and invasive haemodynamic assessment. Europace 2012 Mar;14(3):373-9.

2 de Roest GJ, Allaart CP, Kleijn SA, et al. Prediction of long-term outcome of cardiac resynchronization therapy by acute pressure-volume loop measurements. Eur J Heart Fail 2012 Nov 25.
